# Mosquitoes (Diptera: Culicidae) and mosquito-borne diseases in Mali, West Africa

**DOI:** 10.1186/s13071-018-3045-8

**Published:** 2018-08-13

**Authors:** Fatalmoudou Tandina, Ogobara Doumbo, Alpha Seydou Yaro, Sékou F. Traoré, Philippe Parola, Vincent Robert

**Affiliations:** 1 Aix Marseille Univ, IRD, AP-HM, SSA, VITROME, IHU-Méditerranée Infection, Marseille, France; 2 Department of Epidemiology of Parasitic Diseases, Malaria Research and Training Center, Faculty of Sciences and Techniques, University of Science, Techniques and Technologies of Bamako, Bamako, Mali; 3 MIVEGEC Unit, IRD-CNRS-Univ. Montpellier, Montpellier, France

**Keywords:** *Aedes*, *Anopheles*, *Culex*, Anophelinae, Culicinae, Vector

## Abstract

Mosquito-borne diseases cause major human diseases in almost every part of the world. In West Africa, and notably in Mali, vector control measures help reduce the impact of mosquito-borne diseases, although malaria remains a threat to both morbidity and mortality. The most recent overview article on mosquitoes in Mali was published in 1961, with a total of 88 species. Our present review focuses on mosquitoes of medical importance among which the *Anopheles* vectors of *Plasmodium* and filaria, as well as the *Culex* and *Aedes* vectors of arboviruses. It aims to provide a concise update of the literature on Culicidae, covering the ecological areas in which the species are found but also the transmitted pathogens and recent innovative tools for vector surveys. This review highlights the recent introduction of invasive mosquito species, including *Aedes albopictus* and *Culex neavei*. The comprehensive list of mosquito species currently recorded includes 106 species (28 species of the Anophelinae and 78 species of the Culicinae). There are probable gaps in our knowledge concerning mosquitoes of the subfamily Culicinae and northern half of Mali because most studies have been carried out on the genus *Anopheles* and have taken place in the southern part of the country. It is hoped that this review may be useful to decision makers responsible for vector control strategies and to researchers for future surveys on mosquitoes, particularly the vectors of emerging arboviruses.

**Electronic supplementary material:**

The online version of this article (10.1186/s13071-018-3045-8) contains supplementary material, which is available to authorized users.

## Background

Mosquito vectors can transmit several pathogens, including arboviruses, protozoans and filariae that cause infectious diseases of significant public health concern [1]. To a lesser extent, they may also transmit bacterial diseases [2]. Mosquitoes of medical importance belong to the family Culicidae and are widely distributed around the world. This large family currently encompasses 3556 valid species [3] of mosquitoes distributed within the subfamilies Culicinae and Anophelinae [4]. The mosquito vectors mainly belong to three genera, *Anopheles*, *Aedes* and *Culex.*

*Anopheles* mosquitoes have been continuously studied in Mali since 1906. The first detailed work on mosquitoes in the French Sudan (former name of Mali) has been carried out by Le Moal [5] and Bouffard [6]. Since then, several studies have contributed towards our understanding of this subject, but until 1950 there were only twelve known mosquito species in the country: eight *Anopheles* spp., one *Aedes* sp., two *Culex* spp. and one *Mansonia* sp. (Table 1). These data illustrate the distribution of *Anopheles* along the Niger River, with some information on their preimaginal development sites and adult resting places. In 1961, Hamon et al. [7] considerably improved the catalog of mosquitoes, taking into account previous works and personal observations. Their list contained 88 mosquitoes, including 20 species of the Anophelinae and 68 species of the Culicinae (Table 1). Nevertheless, many of them only existed in a few places, because the northern half of the country had not yet been surveyed. Among the *Anopheles* species, Hamon et al. [7] recognized *Anopheles* (*Cellia*) *gambiae*, *An.* (*Cellia*) *funestus* and *An.* (*Cellia*) *nili* as the main malaria vectors.

**Table 1:** List of Culicidae species reported in Mali since 1908

Subfamily	Species				
	1908 [5]	1950 [7]	1961 [7]	Recent (2000-present)	Reference (2000-present)
Anophelinae			*An. arabiensis*	*An. arabiensis*	[72, 88–90]
			*An. brohieri*		
				*An. brunnipes*	[41, 89]
				*An. coluzzii*	[89, 90]
		*An. coustani*	*An. coustani*		
				*An. domicola*	[41, 89]
			*An. flavicosta*		
		*An. funestus*	*An. funestus*	*An. funestus*	[41, 89]
	*An. gambiae*	*An. gambiae*	*An. gambiae*	*An. gambiae*	[72, 89, 90]
			*An. hancocki*		
			*An. leesoni*		
			*An. longipalpis*		
			*An. maculipalpis*		
				*An. maliensis*	[61]
		*An. nili*	*An. nili*		
			*An. obscurus*		
		*An. paludis*	*An. paludis*		
		*An. pharoensis*	*An. pharoensis*		
			*An. pretoriensis*		
			*An. rhodesiensis rhodesiensis*		
			*An. rivulorum*		
		*An. rufipes*	*An. rufipes rufipes*	*An. rufipes broussesi*	[89]
				*An. sergentii sergentii*	[41]
				*An. sergentii macmahoni*	[61]
				*An. schwetzi*	[61]
				*An. somalicus*	[61]
			*An. squamosus*		
			*An. wellcomei wellcomei*		
		*An. ziemanni*	*An. ziemanni*	*An. ziemanni*	[41]
Culicinae			*Ad. africana*		
			*Ad. furfurea*		
	*Ae. aegypti*	*Ae. aegypti*	*Ae. aegypti*	*Ae. aegypti*	[6, 72, 89]
				*Ae. albopictus*	[65]
			*Ae. africanus*		
			*Ae. argenteopunctatus*		
			*Ae. cumminsii*		
			*Ae. circumluteolus*		
			*Ae. dalzieli*		
				*Ae. dialloi*	[91]
			*Ae. fowleri*	*Ae. fowleri*	[72]
			*Ae. furcifer*		
			*Ae. grahamii*		
			*Ae. haworthi*		
			*Ae. hirsutus*		
			*Ae. lineatopennis*		
			*Ae. luteocephalus*		
			*Ae. longipalpis*		
			*Ae. mattinglyi*		
			*Ae. metallicus*		
			*Ae. minutus*		
			*Ae. mucidus*		
			*Ae. mixtus*		
			*Ae. ochraceus*		
				*Ae. opok*	[63]
			*Ae. punctothoracis*		
			*Ae. scatophagoides*		
			*Ae. simpsoni* (*s.l.*)		
			*Ae. stokesi*		
				*Ae. sudanensis*	[64]
			*Ae. tarsalis*		
			*Ae. taylori*		
			*Ae. vittatus*		
			*Cq. aurites*		
			*Cq. maculipennis*		
			*Cq. metallica*		
			*Cx. albiventris*		
			*Cx. annulioris*		
			*Cx. antennatus*		
			*Cx. argenteopunctatus*		
			*Cx. bitaeniorhynchus*	*Cx. bitaeniorhynchus*	[61]
			*Cx. cinereus*		
			*Cx. decens*		
			*Cx. duttoni*		
			*Cx. grahamii farakoensis*		
			*Cx. grahamii grahamii*		
	*Cx. guiarti*		*Cx. guiarti*		
			*Cx. horridus*		
			*Cx. inconspicuosus*		
			*Cx. insignis*		
			*Cx. invidiosus*		
			*Cx. macfìei*		
			*Cx. nebulosus*		
				*Cx. neavei*	[72]
				*Cx. perexiguus*	[72]
			*Cx. perfuscus*		
			*Cx. poicilipes*		
			*Cx. quasiguiarti*		
	*Cx. quinquefasciatus*		*Cx. quinquefasciatus*	*Cx. quinquefasciatus*	[43, 72, 88]
			*Cx. simpsoni*		
			*Cx. trifoliatus*		
			*Cx. univittatus*		
			*Cx. weschei*		
			*Cx. wigglesworthi*		
			*Er. dracaenae*		
			*Fi. uniformis*		
			*Mi. splendens*		
			*Mi. mimomyiaformis*		
			*Mi. plumosa*		
			*Mi. mediolineata*		
			*Ma. africana*		
	*Ma. uniformis*		*Ma. uniformis*		
			*Tr. viridibasis*		
			*Tr. brevipalpis conradti*		
			*Ur. balfouri*		
			*Ur. chorleyi*		
			*Ur. ornata*		
			*Ur. mashonanensis*		
			*Ur. fusca*		

Subsequently, Touré et al. [8–10] carried out studies on the sensitivity of *An. gambiae* (*s.l.*) and *An. funestus* to insecticides and the rates of infection with malaria parasites and filariae in the *An. gambiae* complex. Malaria epidemiological studies have been conducted by Doumbo et al. [11] in the Malian Sahel to fill the data gap on malaria in that region. The results showed that circulation of the malaria parasites takes place through two main vectors, *Anopheles gambiae* (*s.s.*) (chromosomal form Mopti) and *Anopheles* (*Cel.*) *arabiensis* in northern Mali. *Anopheles gambiae* (*s.s.*) was the only vector found in the far north of the country [12]. Several studies have been conducted on the *An. gambiae* complex, including *An. arabiensis* and chromosomal forms of *An. gambiae* (*s.s.*) targeting the differences in their human blood index (anthropophilic rate) as well as spatial and seasonal distributions [13–16].

**Fig. 1:**
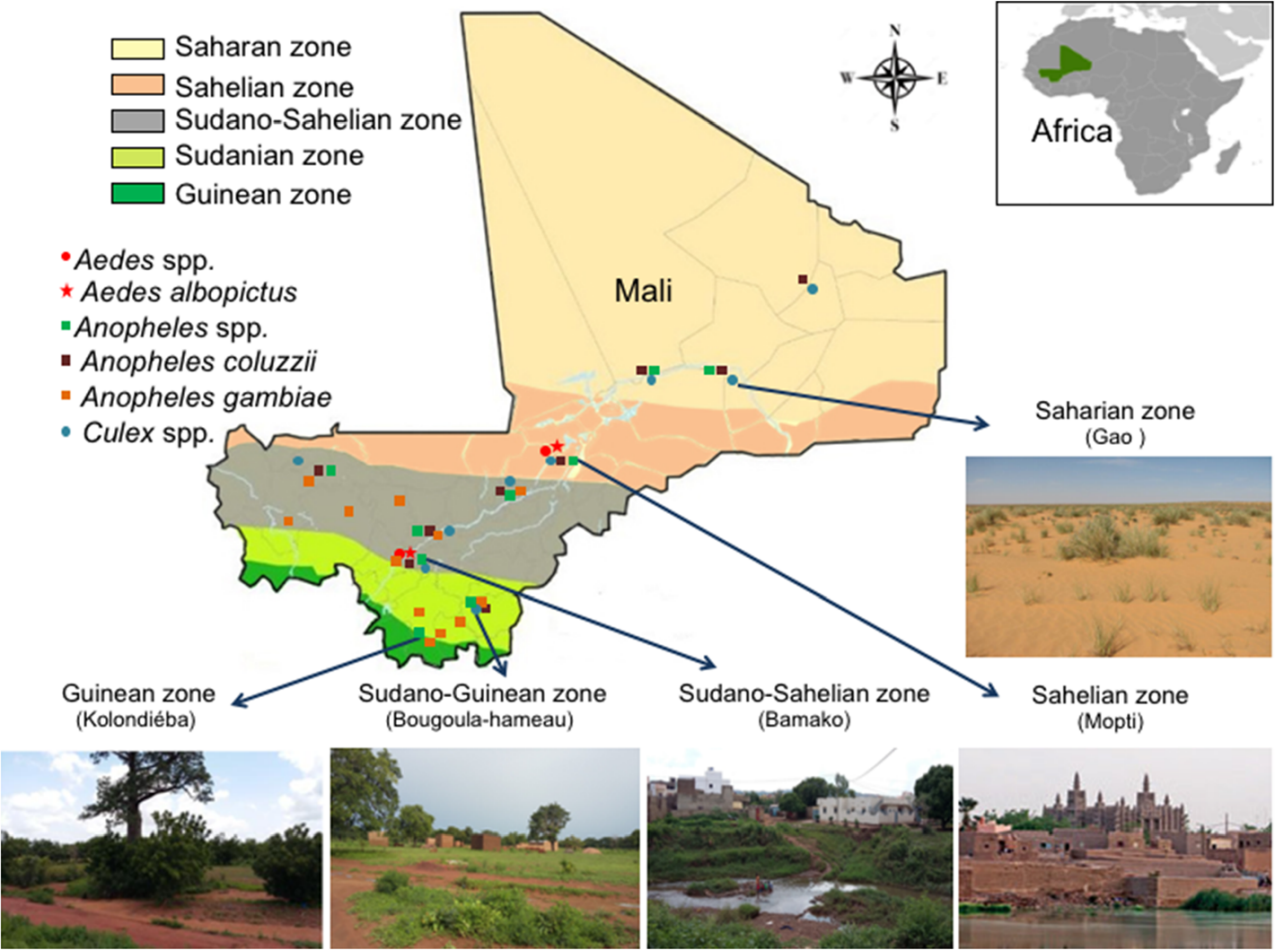
Eco-climatic areas and mosquito distribution in Mali. From north to south, there are five zones including the Saharan zone, the Sahelian zone, the Sudano-Sahelian zone, the Sudanese zone and the Guinean zone. The distribution of some vector mosquitoes is reported, including: *Aedes* spp., *Aedes albopictus*, *Anopheles* spp., *Anopheles coluzzii*, *Anopheles gambiae* and *Culex* spp.

The eco-climatic areas are classified into five facies, i.e. from north to south: the Saharan zone, the Sahelian zone, the Sudano-Sahelian zone, the Sudanian zone and finally, the Guinean zone (Fig. 1) [17]. In the different eco-climatic areas, the human malaria caused by *Plasmodium* spp. continues to be responsible for deaths every year in Mali. This situation is not new as the country has a long history of malaria as the leading cause of morbidity and mortality, mainly among children under five and pregnant women [18].

In order to address this public health problem, free mass distribution of long-lasting insecticide-treated mosquito nets (ITNs) has been introduced by the country’s public health services, mainly for these two at-risk populations [18, 19]. Despite these control measures, malaria remains endemic with 748 deaths in 2000 and 1544 in 2015 [20, 21]. *Anopheles coluzzii*, *An. gambiae* (*s.s.*), *An. arabiensis* and *An. funestus* mosquitoes are the dominant vector species of the *Plasmodium* parasites, including *P. falciparum*, *P. vivax*, *P. malariae*, *P. ovale wallikeri* and *P. ovale curtisi* [4, 20, 22].

Lymphatic filariasis (LF) is mosquito-borne neglected tropical disease and was considered as a public health problem [23]. Lymphatic filariasis, due to the *Wuchereria brancrofti*, has the same anopheline vectors as malaria [4, 24, 25]. It should be noted that since the inception of the Global Programme for the Elimination of Lymphatic Filariasis (GPELF), remarkable progress has been made in this country [23, 26]. Indeed, new cases are not reported indicating an interruption of the transmission [23, 27].

The genus *Aedes* contains several vector species of arboviruses, including yellow fever, dengue, chikungunya, Rift Valley fever and Zika viruses, being responsible for public health problems around almost the entire world [28–30]. Several arboviruses have been reported as being responsible for mortality and morbidity in the country [31, 32]. Three outbreaks of yellow fever have been recently reported, notably in the Sudano-Sahelian area in 1987 (Kati and Kita districts), in 2004 (Kita district), and in 2005 (Bafoulabé district). In these southern parts of the country, 58 cases and 25 deaths occurred [32, 33]. Yellow fever is a hemorrhagic fever transmitted to humans by several species of *Aedes* mosquitoes including *Ae.* (*Diceromyia*) *furcifer* and *Ae.* (*Stegomyia*) *aegypti* [32, 33]. Rift Valley fever is a viral hemorrhagic fever affecting both humans and ruminants, and is an emerging disease which is transmitted by several mosquitoes from different genera, including *Culex*, *Aedes* and *Anopheles* [34–36]. Transmission of Rift Valley fever virus by *Aedes* may be trans-ovarian. Recently, an outbreak of Rift Valley fever was reported in western Niger, near the border with Mali [34, 37].

The genus *Culex* contains several potential vectors of pathogens such as microfilaria *Wuchereria bancrofti* and flaviviruses [38–40]. A number of mosquito-borne disease control measures have now been developed. The most effective are those directed against the mosquitoes, including long-lasting insecticidal nets and indoor residual spraying [20]. For mosquito control measures to be effective, it is necessary to deepen our knowledge of the resting behavior of vector species.

The purpose of this review is to provide an update of the current literature on the Malian Culicidae fauna, covering the ecological areas where they are distributed and to describe the potential pathogens transmitted. Special attention is given to mosquito vectors and the main bio-ecological characteristics of the mosquito species are detailed for the vector species only. Table 2 presents an overview of arboviruses detected in Mali. We also mention the progress made in vector mosquito control and recent innovative tools for mosquito species identification.

**Table 2:** Mosquito-borne arboviruses in Mali

Virus	Source of detection	Vertebrate host	Vector host	Reference
Yellow fever	Patient serum, mosquitoes	Primates	*Aedes* spp.	[32, 33, 92, 93]
Dengue	Patient serum	Primates	*Aedes* spp.	[31, 93]
Chikungunya	Patient serum	Primates, birds, cattle and rodents	*Aedes* spp.; *Culex* spp.	[31, 92, 93]
Zika	Patient serum	Primates	*Aedes* spp.	[92, 93]
Rift Valley fever	Patient serum	Cows, sheep, camels, goats and other mammals	*Aedes* spp.; *Anopheles* spp.; *Culex* spp.	[34–37, 93]
West Nile	Patient serum	Birds, horses and other mammals	*Culex* spp.	[31, 92, 93]

The complete checklist of mosquitoes currently recorded in Mali includes 106 species (3 of them have 2 subspecies each), grouped into the Anophelinae (28 species) and Culicinae (78 species) (Table 1, Additional file 1: Table S1).

## The subfamily Anophelinae: *Anopheles* vectors

The subfamily Anophelinae comprises 488 officially recognized species. Of these, 60 species are important in the epidemiology of malaria, lymphatic filariasis and arboviruses [4]. In Mali, only species of the genus *Anopheles* are present. Twenty-eight *Anopheles* spp. recorded in various entomological surveys are listed in Additional file 1: Table S1 [7, 41–43].

*Anopheles gambiae* (*s.l.*), *An. funestus* and *An. nili* are the main malaria vectors [22, 44]. *Anopheles gambiae* populations have shown a considerable heterogeneity in the country [42, 45–47]. *Anopheles gambiae* (*s.l.*) includes 8 valid species, of which *An. arabiensis, An. coluzzii* and *An. gambiae (s.s.*) are distributed in Mali and are important malaria vectors in this country. The molecular tools used to investigate *An. gambiae* (*s.l.*) have enabled two molecular forms to be differentiated, M and S. The molecular form M refers to the chromosomal form Mopti and was recently named *An. coluzzii* by Coetzee et al. [48]. The molecular form S retains its original name, *An. gambiae* (*s.s*.). This molecular form combines two chromosomal forms known as Savanna and Bamako. These three taxa (*An. coluzzii*, *An. gambiae* chromosomal form Savanna, and *An. gambiae* chromosomal form Bamako) are spread according to the ecoclimatic facies of the country [42, 45–47].

**Fig. 2:**
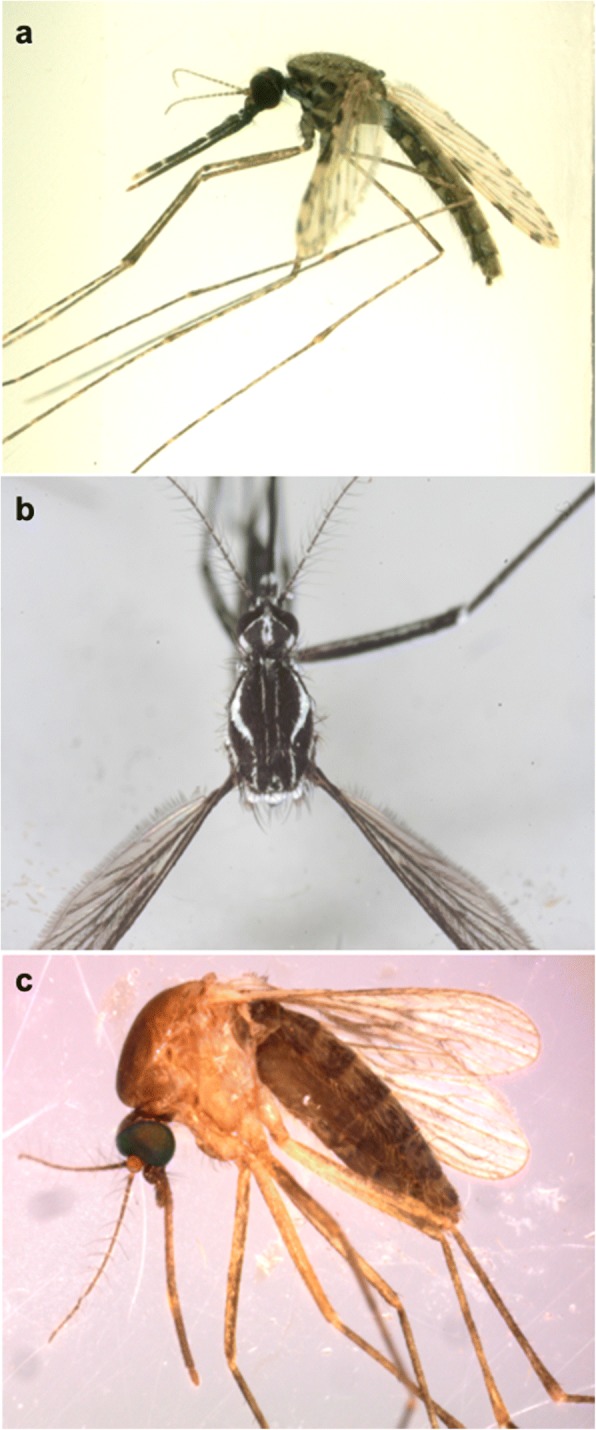
Pictures of three species of mosquitoes that are potential vectors in Mali*.* **a** *Anopheles coluzzii* female (laboratory-reared). **b** *Aedes aegypti* female collected in Gabon. **c** *Culex quinquefasciatus* female collected in Mali

*Anopheles coluzzii* (Fig. 2a) is found in the northern Savanna, in the Sahel, and in the irrigated areas of the inner Niger River delta; it is the most abundant species of the *An. gambiae* complex in the country. Meanwhile, the Savanna chromosomal form is present in the Sudano-Sahelian and Sudano-Guinean areas, particularly in the Kayes and Sikasso regions [47]. The chromosomal form of Bamako is limited to the Sudano-Sahelian zone, particularly around Bamako and in the Sudano-Guinean zone west of Sikasso. The hybrids and recombinants between the Bamako and Coluzzii forms are limited to the Kayes region in western Mali [47].

*Anopheles coluzzii* and *An. gambiae* (*s.s.*) are considered highly anthropophilic and bite humans easily, mainly indoors (endophagic) but also outdoors (exophagic). The main biting activity occurs at night, and after blood-feeding, females leave (exophilic) or remain (endophilic) in these homes [49]. These species have a short larval development period and are often found in larval habitats associated with human activity. Immature stages of *An. coluzzii* develop in permanent or subpermanent larval settings, such as the central Niger River delta and irrigated rice fields. Immature stages of *An. gambiae* (*s.s.*) develop in sunny fresh water without raised vegetation [4, 50] and develop in temporary water such as puddles and ponds. *Anopheles* larval development is optimal six weeks after rice transplantation in the field [42]. Females usually take their first blood meal 24 hours after emergence and a high proportion feed again the same night after oviposition. The dispersal capacity of the females is low; they are usually found between one and three kilometers from their larval sites [1]. However, recent studies have demonstrated the potential ability of *An. coluzzii* to migrate over long distances and aestivate [51, 52]. Thus, *An. gambiae* (*s.s.*) spreads across several biotope types which leads to the species being widely distributed. The majority of mosquitoes collected in the Sudan-Savanna ecological zone (southern Mali) consist of the sister taxa, *An. gambiae* (*s.s.*) and *An. coluzzii* [45]. Both species are present in the two Savanna sites, arid Savanna and humid Savanna; however, *Anopheles coluzzii* is predominant in the arid Savanna, and *An. gambiae* (*s.s.*) is predominant in the humid Savanna [45]. Temperature and moisture play an important role in the density of mosquitoes in the ecological areas [53]. *Anopheles gambiae* (*s.s.*), *An. coluzzii* and *An. arabiensis* are the main species represented in all collections in the various ecological areas [45, 54].

*Anopheles arabiensis* is considered to be a species living in dry savannah-like environments. This species is described as anthropophilic and zoophilic; when domestic animal hosts are available, these females prefer to feed on livestock. Furthermore, the *An. arabiensis* species is more likely to prefer the outside environment for feeding (exophagic) and rest for digestion of blood meals (exophilic) [1, 50]. Moreover, blood-feeding usually occurs during the night. The larval habitats are similar to those of *An. gambiae* (*s.s.*), generally temporarily sunny freshwater and permanent water such as rice paddies [4, 50]. *Anopheles gambiae* (*s.l.*) and *An. funestus* are collected both from irrigated and non-irrigated zones [55]. In these areas, *An. gambiae* (*s.l.*) is more abundant than *An. funestus* [55]. However, the densities of both vectors are dynamic and are seasonally dependent. For instance, in recent decades *An. funestus* was more abundant than *An. gambiae* in non-irrigated areas during the cold dry season; in contrast, in the irrigated area during the rainy season, *An. gambiae* (*s.l.*) was found to be more abundant than *An. funestus* [55]. In addition, these mosquito species have also been collected in the rice cultivation area of Office du Niger, located in the inner delta of the Niger River, in the Sudano-Sahelian area [56]. However, a number of recent studies conducted in non-irrigated areas have revealed a significant density of *An. gambiae* complex, compared to *An. funestus* in all seasons [57].

The *An. funestus* subgroup contains six species, including *An. aruni*, *An. confusus, An. funestus* (*s.s.*), *An. parensis*, *An. vaneedeni* and *An. longipalpis*, but only *An. funestus* (*s.s.*) is associated with malaria transmission as a vector [3, 58, 59]. A typical larval habitat for *An. funestus* is a permanent and semi-permanent water body with emergent vegetation. These larvae are found in marshes, large sunny ponds, lake shores and rice fields [42, 60]. *Anopheles funestus* is considered to be highly anthropophilic [60] as the *An. gambiae* complex [57]. These females usually feed indoors (endophagic) after sunset, with a peak occurring during the second half of the night. After feeding, they rest indoors (endophilic) on the upper part of the walls. In many areas, *An*. *funestus* live inside homes, making them vulnerable to residual insecticide control measures [1].

Recently *An. macmahoni* has been considered as being a subspecies of *An. sergentii* [61]. *Anopheles sergentii macmahoni* has never been found biting humans and has no known medical importance [50]. *Anopheles sergentii sergentii* is known as the oasis vector or the desert malaria vector due to its distribution within oases across the Saharan belt [50]. This species was collected in Saharan area [41]. The larval habitats are streams, seepages, canals, irrigation channels, springs, rice fields and most other shallow, unpolluted sites that contain fresh water with slow current, light emergent vegetation or algae [50]. *Anopheles sergentii sergentii* females are described as anthropophilic and zoophilic because they bite both humans and animals. However, this species prefer to feed indoors (endophagic) and sometimes rest for digestion of blood meal (semi-endophilic) [41].

## The subfamily Culicinae

The subfamily Culicinae includes several tribes, including Aedeomyiini, Aedini, Culicini, Ficalbiini, Mansoniini, Toxorhynchitini and Uranotaeniini. A total of 78 species of the Culicinae have been recorded in various entomological surveys and are listed in Table 1 and Additional file 1: Table S1*.*

In this review, we present in detail only species of the genera *Aedes* and *Culex*, because the potential vectors in Mali belong to these two genera.

### *Aedes* as potential vectors in Mali

*Aedes* mosquitoes are dominant vectors of most arboviruses that infect humans and animals worldwide and in West Africa [62]. The distribution of the *Aedes* mosquitoes and the disease they transmit depend on the ecological conditions of each area. Hamon et al. [7] reported the existence of 28 *Aedes* species (Table 1). Two more *Aedes* species, *Ae. opok* [63] and *Ae. sudanensis* [64], were then reported in Mali. Recently, Muller et al. [65] conducted an entomological survey and recorded *Ae.* (*Stegomyia*) *albopictus*. According to Hamon et al. [7] and Muller et al. [65], the potential vectors of arboviruses are *Ae. aegypti*, *Ae. albopictus*, *Ae.* (*Stegomyia*) *africanus*, *Ae. furcifer*, *Ae.* (*Aedimorphus*) *fowleri*, *Ae.* (*Stegomyia*) *luteocephalus* and *Ae.* (*Aedimorphus*) *ochraceus* [7, 65].

*Aedes aegypti* (Fig. 2b) and *Ae. albopictus* are the major vectors of the dengue virus (DENV). Of the four viral serotypes of DENV, three (serotypes 2, 3 and 4) are present in West Africa, particularly at the border between Burkina Faso and Mali [66, 67]. In Mali, epidemic monitoring of DENV is crucial because the *Aedes* vectors are present and patient serum samples were positive for this viral infection [7, 31, 65]. The chikungunya virus (CHIKV) belongs to the family *Togaviridae* and genus *Alphavirus*. The CHIKV has the same vectors as DENV and circulates in population at risk of epidemic [7, 31, 65].

*Aedes aegypti* is the main vector of the yellow fever virus (YFV) and is the only domestic vector of this virus in West Africa [62]. In Mali*,* this species has been reported in towns, villages as well as in natural wooded savannas. Their breeding water is mostly clean or has a moderate content of organic matter. Females lay their eggs in tree holes and artificial containers such as tires, flower pots, cisterns and abandoned containers, increasing the risk of urban YFV epidemics in Mali [7]. *Aedes aegypti* eggs are resistant to desiccation for up to one year and are able to maintain vertical transmission, allowing them to be constantly present during the dry season and to be transported passively [68]. At a favourable temperature, larvae hatch two days after laying, pupation occurs after eight days and adults emerge between nine and ten days after laying. Females bite during the day in the shade or darkness, with activity peaks in the morning and late afternoon, after feeding, they rest indoors (endophilic). They appear to prefer human blood to that of domestic animals [1].

*Aedes albopictus* eggs are resistant to desiccation and can survive for several months [68]. Their passive transport has allowed this species to invade several continents, although it is of Asian origin. This invasion is linked to their great ecological and physiological plasticity, which allows them to adapt to new biotopes [68]. Their longevity is around 30 days for females and 18 days for males, under favourable temperature conditions. In 2016, the first identification of *Ae. albopictus* in two areas (Mopti and Bamako along the Niger River) stressed the need to monitor mosquitoes [65]. These females usually bite humans and other mammalian vertebrates such as rabbits, dogs, cows, squirrels and, occasionally, avian hosts. Their opportunistic trophic preferences make them potentially capable of transferring infectious agents from animals to humans [68]. This species is exophagic during the day in umbrageous areas and endophagic at sunset and during the night [1]. Egg-laying females are attracted to containers, buckets, plastic bags, used tires and fishing boats to lay their eggs. Interestingly, all these habitats are created by humans [65].

*Aedes furcifer* is a sylvatic vector of YFV and DENV. This species was implicated in the yellow fever outbreak that occurred in two Sudano-Sahelian areas in the Kati and Kita districts in 1987, in Mali [33]. Their larval sites are primarily tree holes and secondary puddles on the roadside [7].

Finally, at least one species of the *Ae. simpsoni* complex was recorded in Mali. It remains unclear if this species is *Ae. bromeliae* or *Ae. lilii* or if both are present, but it is probably not the nominal species *Ae. simpsoni* (*s.s.*), distributed only in southern Africa [69, 70].

### *Culex* as potential vectors in Mali

The genus *Culex* contains 769 species distributed worldwide [68, 71]. In Mali, 28 *Culex* species (or subspecies) belonging to four subgenera have been recorded (Additional file 1: Table S1) [7, 43, 71, 72]. Among them, *Cx.* (*Oculeomyia*) *poicilipes*, *Cx.* (*Culex*) *antennatus*, *Cx.* (*Culex*) *quinquefasciatus* and *Cx.* (*Culex*) *neavei* are potential vectors of *Flavivirus* and lymphatic filariasis [7, 35, 43, 71–75]*. Culex* females lay their eggs on permanent or temporary water surfaces, their larval habitats are ponds, flooded ground, irrigated crops, river banks and tree holes [68]. *Culex quinquefasciatus* (Fig. 2c) is a member of the *Cx.* (*Culex*) *pipiens* complex and is the most abundant species in tropical Africa. *Culex quinquefasciatus* is widely distributed in West Africa and is an important vector of *Wuchereria bancrofti* [24, 71]*. Culex quinquefasciatus* larvae have been reported in several types of habitat, including clear water, brackish, polluted water with organic matter and human waste, ditches, sewage, latrines and artificial containers [1]. Females bite humans and domestic animals such as poultry, dogs, cats and pigs. They are endophagic during the night and exophagic from sunset to dawn [1]. They rest indoors (endophilic) following their blood meals [68].

We recently conducted entomological surveys using classical and innovative tools in order to identify mosquitoes, such as molecular techniques and MALDI-TOF MS (see below). This allowed us to update the list of mosquito vectors in Mali by describing new mosquito species. We reported for the first time *Cx. neavei* and *Cx. perexiguus* [72]. *Culex neavei* species has been identified in three sites and is considered a potential vector of WNV on the border between Senegal and Mali [43, 74]. Other authors have shown that this species is a potential vector of WNV and USUV [75].

*Culex poicilipes* is considered a potential vector of Rift Valley Fever Virus (RVFV) in Barkedji, Senegal [35] and this mosquito species is abundant throughout Mali [7]. The larval habitats of this species include streams, flooded meadows, swamps, ponds and irrigated farmland along the Niger River that could increase the risk of transmission of Rift Valley fever. Furthermore, the virus is circulating on the border between Mauritania and Mali, as well as in western Niger [37, 76].

## Strategies for mosquito vector control

National malaria control programmes, in collaboration with the WHO, have encouraged the use of mosquito nets impregnated with long-lasting insecticide and indoor residual spraying. These efforts have contributed to a decrease in malaria cases in Mali [20]. There are four classes of insecticides recommended by the WHO, namely pyrethroids, organochlorines, organophosphates and carbamates. Mosquitoes have become resistant to a number of these insecticides, posing a serious threat to the success of vector control programmes [20].

Researchers have reported *An. gambiae* (*s.l.*) resistance mecanisms to several insecticides, including dichlorodiphenyltrichloroethane (DDT), deltamethrin (PY), lambda-cyhalothrin (PY), bendiocarb (CA) and fenitrothion (OP) [77]*.* The mutation on two target sites (*kdr* and *ace*-*1R*) and the metabolic detoxification systems (monooxygenases and esterases) have been identified in *An. coluzzii*, *An. gambiae* (*s.s.*) and *An. arabiensis* [77].

The attractive toxic sugar bait (ATSB) plant-spraying methods against *An. gambiae* have reduced the density and longevity of this vector, suggesting that ATSB’s plant spraying methods can be used as a new tool to control this species [78]. Recently, Lin Zhu et al. [79] confirmed the effectiveness of ATSB on malarial vectors in Africa.

Finally, the entomopathogenic fungus *Beauveria bassiana* treatments significantly reduced the blood-feeding of *Cx. quinquefasciatus* in the field. These results show that *B. bassiana* could be a potential new mosquito control alternative [80].

Larvae control reduces the development of the vector population by using chemical toxins (larvicides), biological toxins or fish predators as biological controls [20]. Although larvicides are useful in some contexts, they are only feasible in areas where most larval sites are easily located, so they are systematically identifiable and can be fully covered by the intervention. This method often has a greater impact on transmission than adulticide methods that reduce both the density (number) of mosquitoes and their lifespan [20].

## Innovative methodologies for mosquito species identification

New diseases and new vectors that colonize new territories, where they were previously absent, are continuously emerging. For example, the tiger mosquito, *Ae. albopictus*, has been found in almost every continent of the world [65, 81]. The mosquito-borne diseases are a major public health problem worldwide. Formal mosquito identification is essential to effectively control vectors. Morphological identification using dichotomous keys is the most widely used method for entomological surveys [49]. Currently, entomologists also use identification keys on a CD-ROM [41]. The limits of morphological identification lay in the distinction between sub-species, in particular the cryptic forms of *An. gambiae* (*s.s.*) [82]. In Mali, molecular methods were used to distinguish *An. gambiae* (*s.l.*) cryptic species and forms and certain species of the *Cx. pipiens* complex, which are difficult to distinguish by their morphology [45, 47, 72, 77]. A limitation of molecular methods is their cost; the comprehensiveness of databases and the overall operating time.

Matrix-Assisted Laser Desorption Ionization-Time of Flight (MALDI-TOF MS) has revolutionized microbiology and is now routinely used. MALDI-TOF mass spectrometry has been used in medical entomology to identify arthropods [83]. This technique has been used in the laboratory for the identification of adult mosquitoes from protein extracts from the legs. Aquatic stages have also been identified, including eggs and larvae [84–86]. MALDI-TOF mass spectrometry is now used during entomological surveys. The preliminary study used field mosquitoes to update the European mosquito catalog [87].

In Mali, MALDI-TOF MS was applied on the mosquitoes and their midgut microbiota collected in the rural area of Bougoula-hameau in the Sikasso region. This technology was used to identify Malian mosquitoes from protein extracts from their legs [43]. In addition of the mosquito identification, their blood meal sources were also determined using MALDI-TOF MS. Specimens collected from three regions in the Sudan-Savanna area (around urban Bamako, the rural area of the Sikasso region and the rural area around Kati) in Mali [72]. In this country, eight mosquito species have been identified, namely *An. gambiae* (*s.s.*), *An. coluzzii*, *An. arabiensis*, *Cx. quinquefasciatus*, *Cx. neavei*, *Cx. perexiguus*, *Ae. aegypti* and *Ae. fowleri* [72]. Indeed, the mosquito blood-meal sources were correctly identified and matched with the blood of human, chicken, cow, donkey, dog and sheep. Thus, this innovative tool successfully identified Malian mosquitoes as well as their blood-meal sources and enabled the first detection of two mosquito species in Mali, *Cx. neavei* and *Cx. perexiguus* [72].

## Conclusions

Recent collections of mosquitoes in Mali focus mainly on vector species involved in the transmission of infectious diseases that cause a public health problem. In this context, the recent publications only provide information on the ecology, distribution and associated pathogens of *Anopheles*, *Aedes* and *Culex* vectors. We believe that these gaps may be due to collection techniques and their relevance to public health. Indeed, a large number of vectors belonging to the family Culicidae have been identified, including *Ae. aegypti*, *Ae. albopictus*, *An. coluzzii*, *An. gambiae* (*s.s.*), *An. arabiensis*, *An. funestus* (*s.s*.), *Cx. poicilipes*, *Cx. antennatus*, *Cx. quinquefasciatus* and *Cx. neavei* species. They are potential vectors for a number of arboviral, protozoan and filarial pathogens. Our review has contributed to updating the current literature on mosquitoes and mosquito-borne diseases in Mali. This review may be necessary for a future nationwide entomological field surveys for mosquito vector controls.

## Additional file

Additional file 1: **Table S1.** List of mosquitoes reported in Mali, West Africa. (PDF 92 kb)

## Abbreviations

ATSB: Attractive toxic sugar bait
CHIKV: Chikungunya virus
DENV: Dengue virus
GNTs: Glue net traps
GPELF: Global Programme for the Elimination of Lymphatic Filariasis
LF: Lymphatic filariasis
MALDI-TOF MS: Matrix-Assisted Laser Desorption Ionization Time-Of-Flight Mass Spectrometry
MRTC: Malaria Research and Training Center
RVFV: Rift Valley fever virus
USUV: Usutu virus
VITROME: Vecteurs Infections Tropicales et Mediterranéennes
WHO: World Health Organization
WNV: West Nile virus
YFV: Yellow fever virus
