# Supplementary material for: Mosquitoes (Diptera: Culicidae) and mosquito-borne diseases in Mali, West Africa
Source: Parasit Vectors. 2018 Aug 13;11:467. doi: 10.1186/s13071-018-3045-8 (PMC6090629; doi:10.1186/s13071-018-3045-8)
Supplement: Supplementary file 1 — Table S1. List of mosquitoes reported in Mali, West Africa. (PDF 92 kb) [file 13071_2018_3045_MOESM1_ESM.pdf]

Table S1. Mosquito list from Mali, West Africa

Subfamily	Tribe	Genus	Subgenus	Species/subspecies	Descriptor/year	Pathogens	Areas of distribution*	References
**Anophelinae**	/	***Anopheles***	*Anopheles*	*An. coustani*	Laveran, 1900		All	[1,2]
				An. obscurus	(Grünberg, 1905)		G, SG	[1,2]
				An. paludis	Theobald, 1900		Sl, SS	[1,2]
				An. ziemanni	Grünberg, 1902		All	[1,2]
			Cellia	*An. arabiensis*	Patton, 1905	*P. falciparum P. vivax, P. malariae, P. ovale wallikeri, P. ovale curtisi, W. bancrofti*	All	[1–4]
				An. brohieri	Edwards, 1929		G, SG, SS	[1,2]
				An. brunnipes	(Theobald, 1910)		G, SG	[2]
				An. coluzzii	Coetzee & Wilkerson, 2013	*P. falciparum P. vivax, P. malariae, P. ovale wallikeri, P. ovale curtisi, W. bancrofti*	All	[1,2,4]
				An. domicola	Edwards, 1916		G, SG, SS	[2]
				An. flavicosta	Edwards, 1911		G, SG, SS	[1,2]
				An. funestus	Giles, 1902	*P. falciparum, W. bancrofti*	All	[1–4]
				An. gambiae	Giles, 1902	*P. falciparum P. vivax, P. malariae, P. ovale wallikeri, P. ovale curtisi, W. bancrofti*	G, SG, SS	[1,2,4]
				An. hancocki	Edwards, 1929		G, SG	[1,2]
				An. leesoni	Evans, 1931		G, SG, SS	[1,2]
				An. longipalpis	Theobald, 1903		G, SG, SS	[1,2]
				An. maculipalpis	Giles, 1902		G, SG, SS	[1,2]
				An. maliensis	Bailly-Choumara & Adam, 1959		G, SG	[20]
				An. nili	Theobald, 1904	*P. falciparum*	G, SG, SS	[1–4]
				An. pharoensis	Theobald, 1901		All	[1,2]
				An. pretoriensis	(Theobald, 1903)		G, SG, SS	[1,2]
				An. rhodesiensis rhodesiensis	Theobald, 1901		SS	[1,2]
				An. rivulorum	Leeson, 1935		SS	[1,2]
				An. rufipes rufipes	(Gough), 1910		All	[1,2]
				An. rufipes broussesi	Edwards, 1929		All	[20]
				An. sergentii sergentii	(Theobald, 1907)		Sr	[2]
				An. sergentii macmahoni	Evans, 1936		All	[20]
				An. schwetzi	Evans, 1934			[20]
				An. somalicus	Rivola & Holstein, 1957		G, SG	[20]
				An. squamosus	Theobald, 1901		All	[1,2]
				An. wellcomei wellcomei	Theobald, 1904		Sl, SS	[1,2]

**Culicinae**	**Aedeomyiini**	***Aedeomyia***	*Aedeomyia*	*Ad. africana*	Neveu Lemaire, 1906		SS	[1]
			Lepiothauma	*Ad. furfurea*	(Enderlein, 1923)		SS	[1]
	**Aedini**	***Aedes***	*Aedimorphus*	*Ae. cumminsii*	(Theobald, 1903)		SS	[1]
				Ae. dalzieli	(Theobald, 1910)		G, SG, SS	[1]
				Ae. fowleri	(de Charmoy, 1908)		G, SG, SS	[1,5]
				Ae. hirsutus	(Theobald, 1901)		G, SG, SS	[1]
				Ae. mattinglyi	Hamon & Rickenbach, 1954		G, SG	[1]
				Ae. ochraceus	(Theobald, 1901)		G, SG, SS	[1]
			Albuginosus	*Ae. haworthi*	(Edwards, 1923)		G, SG	[1]
				Ae. stokesi	(Evans, 1929)		G, SG	[1]
			Catageiomyia	*Ae. argenteopunctatus*	(Theobald, 1901)		G, SG, SS	[1]
				Ae. dialloi	Hamon & Brengues,1965		G, SG	[6]
				Ae. minutus	(Theobald, 1901)		G, SG, SS	[1]
				Ae. mixtus	Edwards, 1936		G, SG, SS	[1]
				Ae. punctothoracis	(Theobald, 1909)		G, SG, SS	[1]
				Ae. tarsalis	(Newstead, 1907)		G, SG, SS	[1]
			Diceromyia	*Ae. furcifer*	(Edwards, 1913)	YFV	G, SG, SS	[1,7]
				Ae. taylori	Edwards, 1936		G, SG, SS	[1]
			Fredwardsius	*Ae. vittatus*	(Bigot, 1861)		G, SG, SS	[1]
			Mucidus	*Ae. grahamii*	Theobald, 1909		G, SG	[1]
				Ae. mucidus	(Karsch, 1887)		SS	[1]
				Ae. scatophagoides	Theobald, 1901		G, SG, SS	[1]
				Ae. sudanensis	(Theobald, 1908)			[8]
			Neomelaniconion	*Ae. circumluteolus*	(Theobald, 1908)		G, SG, SS	[1]
				Ae. lineatopennis	(Ludlow, 1905)		SS	[1]
			Stegomyia	*Ae. aegypti*	(Linnaeus, 1762)	YFV,DENV,CHIKV	G, Sl, SG, SS	[1,7,9,10]
				Ae. albopictus	(Skuse, 1895)	DENV,CHIKV	Sl, SS	[10,11]
				Ae. africanus	Theobald, 1901		G, SG	[1]
				Ae. luteocephalus	(Newstead, 1907)		G, SG, SS	[1]
				Ae. opok	Corbet & van Someren, 1962		G, SG	[12]
				Ae. metallicus	(Edwards, 1912)		G, SG	[1]
				Ae. simpsoni s.l.	(Theobald, 1905)		SS	[1]
			Zavortinkius	*Ae. longipalpis*	(Grünberg, 1905)		G, SG	[1]
		Eretmapodites	/	*Er. dracaenae*	Edwards, 1916		G, SG	[1]
	**Culicini**	***Culex***	*Culex*	*Cx. antennatus*	(Becker, 1903)		G, SG, SS	[1,3]
				Cx. argenteopunctatus	(Ventrillon, 1905)		G, SG, SS	[1,3]
				Cx. decens	Theobald, 1901	WNV	G, Sr, SG, SS	[1,3,13]
				Cx. duttoni	Theobald, 1901		G, SG, SS	[1,3]
				Cx. guiarti	Blanchard, 1905		G, SG, SS	[1,3]
				Cx. grahamii grahamii	Theobald, 1910		G, SG, SS	[1,3]
				Cx. grahamii farakoensis	Hamon, 1954		G, SG	[1]
				Cx. invidiosus	Theobald, 1901		G, SG, SS	[1,3]
				Cx. neavei	Theobald, 1906	WNV, USUV	G, SG, SS	[5,13–15]

				Cx. perexiguus	Theobald, 1903		G, SG	[3,5]
				Cx. perfuscus	Edwards, 1914		G, SG, SS	[1,3]
				Cx. quasiguiarti	Theobald, 1910		G, SG	[1,3]
				Cx. quinquefasciatus	Say, 1823	*W. bancrofti,* WNV	G, SG, SS	[1,5,13,16,17]
				Cx. simpsoni	Theobald, 1905		G, SG, SS	[1,3]
				Cx. trifoliatus	Edwards, 1914		G, SG	[1,3]
				Cx. univittatus	Theobald, 1901		G, SG, SS	[1,3]
				Cx. weschei	Edwards, 1935		G, SG, SS	[1,3]
			Culiciomyia	*Cx. cinereus*	Theobald, 1901		G, SG	[1,3]
				Cx. macfiei	Edwards, 1923		G, SG, SS	[1,3]
				Cx. nebulosus	Arnell, 1973		G, SG, SS	[1,3]
			Eumelanomyia	*Cx. albiventris*	Edwards, 1922		G, SG	[1,3]
				Cx. horridus	Edwards, 1922		G, SG, SS	[1,3]
				Cx. inconspicuosus	(Theobald, 1908)		G, SG, SS	[1,3]
				Cx. insignis	(Carter, 1911)		G, SG	[1,3]
				Cx. wigglesworthi	Edwards, 1941		G, SG	[1,3]
			Oculeomyia	*Cx. annulioris*	Theobald, 1901		G, SG, SS	[1,3]
				Cx. bitaeniorhynchus	Giles, 1901		G, SG, SS	[20]
				Cx. poicilipes	(Theobald, 1903)	RVFV, WNV	G, SG, SS	[1,18]
	**Ficalbiini**	***Mimomyia***	*Mimomyia*	*Mi. mimomyiaformis*	(Newstead, 1907)		G, Sl, SG, SS	[1]
				Mi. splendens	Theobald, 1903		G, SG, SS	[1]
				Mi. plumosa	(Theobald, 1901)		G, SG, SS	[1]
			Etorleptiomyia	*Mi. mediolineata*	(Theobald, 1904)		SS	[1]
		Ficalbia	/	*Fi. uniformis*	(Theobald, 1904)		G, SG, SS	[1]
	**Mansoniini**	***Coquillettidia***	*Coquillettidia*	*Cq. aurites*	(Theobald, 1901)		G, SG	[1]
				Cq. maculipennis	(Theobald, 1911)		G, SG, SS	[1]
				Cq. metallica	(Theobald, 1901)		G, SG	[1]
		Mansonia	*Mansonioides*	*Ma. africana*	(Theobald, 1901)	*W. bancrofti*	G, SG, SS	[1,19]
				Ma. uniformis	(Theobald, 1901)	*W. bancrofti*	G, SG, SS	[1,19]
	**Toxorhynchitini**	***Toxorhynchites***	*Afrorhynchus*	*Tr. viridibasis*	(Edwards, 1935)		G, SG	[1]
			Toxorhynchites	*Tr. brevipalpis conradti*	Grünberg, 1907		G, SG, SS	[1]
	**Uranotaeniini**	***Uranotaenia***	*Uranotaenia*	*Ur. balfouri*	Theobald, 1904		G, SG, SS	[1]
				Ur. chorleyi	Edwards, 1936		G, SG	[1]
			Pseudoficalbia	*Ur. fusca*	Theobald, 1907		SS	[1]
				Ur. mashonaensis	Theobald, 1901		G, SG, SS	[1]
				Ur. ornata	Theobald, 1909		G, SG	[1]

* Abbreviations for pathogens: ***P:*** *Plasmodium;* **RVFV:** Rift Valley fever Virus; **WNV:** West Nile Virus; W.: *Wuchereria*; **YFV:** Yellow Fever Virus

** Abbreviations for eco-climatic areas: **G:** Guinean; **SG:** Sudano-Guinean; **Sl:** Sahelian; **Sr:** Saharian; **SS:** Sudano-Sahelian

References

1. Hamon J, Eyraud M, Diallo B, Dyemkouma A, Choumara HB, Sylla O. Les moustiques de la République du mali (Diptera: Culicidae). Ann Soc Entomol France 1961;130:95-129.
2. Hervy J, Le Goff G, Geoffroy B, Hervé J, Manga L. Les anophèles de la région Afro-tropicale, logiciel d'identification et d'enseignement. Collection didactique; ORSTOM ed, Paris. 1998.
3. Harbach RE. Classification within the cosmopolitan genus *Culex* (Diptera: Culicidae): the foundation for molecular systematics and phylogenetic research. Acta Trop. 2011;120: 1-14.
4. Hay SI, Sinka ME, Okara RM, Kabaria CW, Mbithi PM, Tago CC, Benz D, Gething PW, Howes RE, Patil AP, Temperley WH, Bangs MJ, Chareonviriyaphap T, Elyazar IR, Harbach RE, Hemingway J, Manguin S, Mbogo CM, Rubio-Palis Y, Godfray HC. Developing global maps of the dominant *Anopheles* vectors of human malaria. PLoS Med. 2010; 7: e1000209.
5. Tandina F, Niaré S, Laroche M, Koné AK, Diarra AZ, Ongoiba A, Berenger JM, Doumbo OK, Raoult D, Parola P. Using MALDI-TOF MS to identify mosquitoes collected in Mali and their blood meals. Parasitology 2018; in press.
6. Hamon J, Brengues J. Observations sur les *Aedes* (*Aedimorphus*) d'Afrique avec description de deux nouvelles espèces : *Ae. lottei n. sp.* et *Ae. dialloi n. sp.* Bull Soc Path exot. 1965; 1:101-108.
7. Cordellier R. L'épidemiologie de la fièvre jaune en Afrique de l'Ouest. Bulletin de l'Organisation mondiale de la Santé 1991; 69(1):73-84.
8. Tyson WH. Notes on African *Aedes,* subgenus *Mucidus* (Diptera: Culicidae). J ent Sot sth Afr. 1970; 33:1.
9. Gardner CL, Ryman KD. Yellow fever: a reemerging threat. Clin Lab Med. 2010; 30:237-260.
10. Vasilakis N, Cardosa J, Hanley KA, Holmes EC, Weaver SC. Fever from the forest: prospects for the continued emergence of sylvatic dengue virus and its impact on public health. Nat Rev Microbiol. 2011; 9:532-541.
11. Muller GC, Tsabari O, Traoré MM, Traoré SF, Doumbia S, Kravchenko VD, Junnila A, Beier JC. First record of *Aedes albopictus* in inland Africa along the River Niger in Bamako and Mopti, Mali. Acta Trop. 2016; 162:245-247.
12. Germain M, Cordellier R, Hervé JP, Geoffroy B, Bouchite B, Ravaonjanahary C, Rickenbach A. Présence en Afrique centrale et occidentale d'*Aedes* (*Stegomyia*) *opok* Corbet et Van Someren - Diagnose différentielle de l'espèce. Cah ORSTOM sér Ent Méd Parasitol. 1975; 1:41-46.
13. Gould E, Pettersson J, Higgs S, Charrel R, de Lamballerie X. Emerging arboviruses: Why today? One Health 2017; 4:1-13.
14. Jupp PG, McIntosh BM, Blackburn NK. Experimental assessment of the vector competence of *Culex* (*Culex*) *neavei* Theobald with West Nile and Sindbis viruses in South Africa. Trans R Soc Trop Med Hyg. 1986; 80:226-230.
15. Nikolay B, Diallo M, Faye O, Boye CS, Sall AA. Vector competence of *Culex neavei* (Diptera: Culicidae) for Usutu virus. Am J Trop Med Hyg. 2012; 86: 993-996.
16. de Souza DK, Koudou B, Kelly-Hope LA, Wilson MD, Bockarie MJ, Boakye DA. Diversity and transmission competence in lymphatic filariasis vectors in West Africa, and the implications for accelerated elimination of *Anopheles*-transmitted filariasis. Parasit Vectors. 2012; 5: 259.
17. Tandina F, Almeras L, Kone AK, Doumbo OK, Raoult D, Parola P. Use of MALDI-TOF MS and culturomics to identify mosquitoes and their midgut microbiota. Parasit Vectors. 2016; 9: 495.
18. Talla C, Diallo D, Dia I, Ba Y, Ndione JA, Morse AP, Diop A, Diallo M. Modelling hotspots of the two dominant Rift Valley fever vectors (*Aedes vexans* and *Culex poicilipes*) in Barkedji, Senegal. Parasit Vectors. 2016; 9: 111.
19. Ughasi J, Bekard HE, Coulibaly M, Adabie-Gomez D, Gyapong J, Appawu M, Wilson MD, Boakye DA. *Mansonia africana* and *Mansonia uniformis* are vectors in the transmission of *Wuchereria bancrofti* lymphatic filariasis in Ghana. Parasit Vectors. 2012; 5: 89.
20. WRBU. The Walter Reed Biosystematics Unit; Gaffigan TV, Wilkerson RC, Pecor JE, Stoffer JA, Anderson T. Systematic Catalog of Culicidae. Smithsonian Institution. http://www.mosquitocatalog.org/ Accession on February 05, 2018.
